# Meckelin 3 Is Necessary for Photoreceptor Outer Segment Development in Rat Meckel Syndrome

**DOI:** 10.1371/journal.pone.0059306

**Published:** 2013-03-13

**Authors:** Sarika Tiwari, Scott Hudson, Vincent H. Gattone, Caroline Miller, Ellen A. G. Chernoff, Teri L. Belecky-Adams

**Affiliations:** 1 Department of Biology, Indiana University-Purdue University Indianapolis, Indianapolis, Indiana, United States of America; 2 Department of Anatomy and Cell Biology, Indiana University School of Medicine, Indianapolis, Indiana, United States of America; 3 Center for Regenerative Biology and Medicine, Indiana University-Purdue University Indianapolis, Indianapolis, Indiana, United States of America; Schepens Eye Research Institute, Harvard Medical School, United States of America

## Abstract

Ciliopathies lead to multiorgan pathologies that include renal cysts, deafness, obesity and retinal degeneration. Retinal photoreceptors have connecting cilia joining the inner and outer segment that are responsible for transport of molecules to develop and maintain the outer segment process. The present study evaluated meckelin (MKS3) expression during outer segment genesis and determined the consequences of mutant meckelin on photoreceptor development and survival in Wistar polycystic kidney disease Wpk/Wpk rat using immunohistochemistry, analysis of cell death and electron microscopy. MKS3 was ubiquitously expressed throughout the retina at postnatal day 10 (P10) and P21. However, in the mature retina, MKS3 expression was restricted to photoreceptors and the retinal ganglion cell layer. At P10, both the wild type and homozygous Wpk mutant retina had all retinal cell types. In contrast, by P21, cells expressing rod- and cone-specific markers were fewer in number and expression of opsins appeared to be abnormally localized to the cell body. Cell death analyses were consistent with the disappearance of photoreceptor-specific markers and showed that the cells were undergoing caspase-dependent cell death. By electron microscopy, P10 photoreceptors showed rudimentary outer segments with an axoneme, but did not develop outer segment discs that were clearly present in the wild type counterpart. At p21 the mutant outer segments appeared much the same as the P10 mutant outer segments with only a short axoneme, while the wild-type controls had developed outer segments with many well-organized discs. We conclude that MKS3 is not important for formation of connecting cilium and rudimentary outer segments, but is critical for the maturation of outer segment processes.

## Introduction

The vertebrate retina is a multi-layered tissue consisting of cell bodies in the, outer nuclear, inner nuclear, and ganglion cell layers. The vertebrate retina contains 2 types of photoreceptors found in the outer nuclear layer; rods and cones. As photoreceptors differentiate, they form 4 specialized compartments; 1) the outer segment, specialized for transduction of photons, 2) the inner segment containing machinery for producing proteins, lipids, and energy, 3) the nuclear region and 4) the synaptic region, necessary for communicating with horizontal and bipolar cells within the retina [1]. Because of this compartmentalization, the sorting of proteins and other components to the right compartment is a highly regulated process in photoreceptors [2].

The inner and outer segments (OS) of photoreceptor cells are joined by a modified non-motile connecting cilium through which essential elements are transported for outer segment morphogenesis. The connecting cilia in the photoreceptor is a “9+0” primary cilia that has nine microtubule doublets without a central pair [3]. The central core of the cilium is held in place by a microtubule backbone called an axoneme that is anchored in the basal body in the inner segment. The connecting cilium uses a specialized system called intraflagellar transport (IFT) as a pathway for the transport of proteins to and from the outer segment [4]. While much information has been accumulated concerning intraflagellar transport, there still remain many questions about the mechanisms of outer segment formation, protein transport through the connecting cilium, and the implications of alterations in protein trafficking to diseases affecting outer segment development and/or maintenance.

Ciliopathies, including Meckel-Gruber syndrome (MKS), are a group of genetic disorders characterized by mutations in proteins found in the primary cilia [5]. MKS is a rare, autosomal recessive, lethal, ciliopathic, genetic disorder characterized by renal cystic dysplasia, and central nervous system malformations, but can also be associated with situs inversus, polydactyl and hepatic developmental defects [6]. MKS or Meckel-like syndrome has been linked to ten genes, the protein products of which are all associated with either the basal body or the cilium [7]–[13]. The Meckelin 3 (MKS3) gene is one of the first to be associated with the Meckel-Gruber syndrome and encodes a 995 amino acid seven pass transmembrane protein with a large extracellular domain that contains topological homology with the WNT family frizzled receptors [14]. While little is known regarding the function of MKS3, it has been shown to interact with inversin, a protein that acts as the molecular switch between WNT canonical and planar cell polarity pathway, as well as other proteins that appear to be involved in intraflagellar transport, such as MKS1, MKS2, nesprin, and actin-binding protein filamin A [15]–[19]. Previous work has suggested that this gene may be critical to cilia function in kidney, liver, and retina. [20], [21].

In this manuscript, we have characterized the expression patterns of meckelin with immunohistochemistry in the differentiating postmitotic and mature rat retina and compared localization of MKS3 with subcellular markers and subset of IFT proteins. Using the Wistar-Wpk rat with a spontaneous mutation in the *rMks3* gene [14], it was previously shown that the formation of the photoreceptor outer segment development was dramatically impaired leading to loss of the photoreceptors [20]. Herein, we found that the retina appeared to have the normal complement of cells at P10. However, photoreceptors underwent rapid degeneration around three weeks of life following a brief period when many transduction proteins appear to be mislocalized to the inner segment, nuclear and synaptic regions. Given the data presented in this manuscript, we postulate that MKS3 is localized at the transition zone between the inner and outer segments and that expression of mutant MKS3 leads to progressive early photoreceptor degeneration.

## Materials and Methods

### WPK rat

Heterozygous Wistar wpk rats were provided by Dr. Nauta (Erasmus Medical Center Rotterdam, Rotterdam, Netherlands). All studies were performed with approval of the IU School of Medicine IACUC Approval MD-3119. Animals were housed at the Indiana University School of Medicine Laboratory Animal Resource Center, and the rats were delivered via cesarean section in order to ensure that the line was pathogen-free [22]. Litters from the heterozygous Wpk/+ crosses were sacrificed at 10 days, 14 days, and three weeks. Rats were anesthetized with sodium pentobarbital (100 mg/kg administered i.p.), and then perfusion fixed with 4% paraformaldehyde in 0.1 M phosphate buffer (pH 7.4). Eyes were dissected free, rinsed twice in 1X phosphate buffered saline (PBS; potassium chloride 200 mg/L, potassium phosphate 200 mg/L, sodium chloride 8.0/L, and sodium phosphate 1.150 g/L), pH 7.5 and placed in 20% sucrose made in 0.1 M phosphate buffer overnight. Rats to be used for α-transducin staining were dark-adapted for 12 hours and fixed for immunohistochemistry as above. Eyes were frozen in a 3∶1 ratio of 20 or 30% sucrose in 0.1 M phosphate buffer to optimal cutting temperature (OCT; VWR, Chicago, IL) solution and stored at −80°C.

### Fluorescent Immunohistochemistry

Ten micron sections were cut with a Leica CM3050 S cryostat placed on Superfrost Plus slides (Fisher Scientific, Pittsburgh, PA) and stored at −80°C until used for immunohistochemistry. For some double-label immunofluorescence with MKS3, tyramide amplification was used according to manufacturer's instructions (Perkin Elmer, Waltham, MA). Briefly, sections were brought to room temperature (RT) for 30 minutes (min), post-fixed in 4% paraformaldehyde for 30 min at RT, permeabilized with methanol for 10 min at RT, washed with 1X PBS for 2min twice, and antigen retrieval performed by incubating in 1.0% sodium dodecyl sulfate in 0.1 M phosphate buffer for 5 min at RT. Sections were then rinsed in 1X PBS for 5 min three times and treated with 1.0% sodium borohydride in 1X PBS for 2 min at RT to reduce autofluorescence. Sections were then blocked with 10% serum in 0.25% triton X-100 in 1X PBS for 1 hour (h), and incubated with primary antibodies diluted in 2% serum diluted in 0.05% triton containing PBS overnight at 4°C. Dilutions and sources of each primary antibody can be found in Table 1. The following day, slides were brought to RT, washed in 1X PBS 2 min three times, incubated with 3% H_2_O_2_ diluted in methanol for 15 min at RT, washed again in 1X PBS 2 min three times, then incubated with biotinylated secondary antibody (Vector labs, Burlingame, VT) diluted in 1X PBS at 1∶2000 for 1h at RT. Sections were subsequently washed in 1X PBS in 2 min twice, incubated with a solution containing streptavidin conjugated to horse radish peroxidase at 1∶1500 and secondary antibody conjugated to alexa fluor (Invitrogen, Eugene, OR) or dylight (to recognize the second antibody used in the double-label; Jackson ImmunoResearch, West Grove, PA) for 1 h at RT. Slides were then washed in Buffer 1 (TNT buffer) 2 min twice at RT, incubated in tyramide at 1∶300 in dilution buffer for 5 min at RT. Finally, slides were washed in Buffer 1 for 2 min twice, 1X PBS 2 min twice, and coverslipped with Prolong Gold antifade with DAPI (Invitrogen). In some cases, sections were incubated for 2 min at room temperature with 0.9 M Hoechst dye (H6024, Sigma-Aldrich, St. Louis, MO), then rinsed briefly with 1X PBS at room temperature, and mounted with aqua poly/mount (Polysciences, Warrington, PA).

**Table 1:** Antibodies.

Antibody Name	Supplier	Catalog Number	Dilution
ABCA4	Santa Cruz (Santa Cruz, CA)	sc-21457	1∶200
Brn3a	Millipore (Temecula, CA)	MAB1585	1∶100
Calbindin-D-28k	Sigma-Aldrich (St. Louis, MO)	CB-955	1∶200
Caspase 3	Cell Signaling (Danvers, MA)	Asp175	1∶12,000
Cep 290	Pro Science (Poway, CA)	46-439	1∶200
Chx10	Exalpha (Shirley, MA)	X1180P	1∶500
CRX	Cheryl Gregory-Evans	NA	1∶500
Cytochrome C	Abcam (Cambridge, MA)	Ab13575	1∶200
GFAP	Dako (Carpinteria, CA)	Z0334	1∶500
Glutamine Synthetase	Millipore (Temecula, CA)	MAB302	1∶250
IFT20	Santa Cruz (Santa Cruz, CA)	sc107627	1∶100
IFT88	Everest biotech (Ramona, CA)	E070610	1∶500
Meckelin	Novus (Littleton, CO)	NBP1-06590	1∶200
Opsin L/M (Red/Green)	Millipore (Temecula, CA)	AB5405	1∶200
Opsin S (Blue)	Millipore (Temecula, CA)	AB5407	1∶200
Parvalbumin	Abcam (Cambridge, MA)	ab11427	1∶2,000
Parvalbumin	Sigma-Aldrich (St. Louis, MO)	P3088	1∶2,000
Rhodopsin	Millipore (Temecula, CA)	MAB5356	1∶250
Sox2	Santa Cruz (Santa Cruz, CA)	sc-17320	1∶250
Rod Transducin	Santa Cruz (Santa Cruz, CA)	sc-389	1∶200
Tubulin	Sigma-Aldrich (St. Louis, MO)	T 6793	1∶4,000

Some slides were labeled using conventional indirect immunofluorescence. These slides were treated identically to slides immunolabeled using tyramide amplification up through the incubation with the primary antibody. Following primary antibody incubation, slides were washed 10 min in 1X PBS twice, then incubated for 1 h with the appropriate fluorescent secondary antibody. Slides were subsequently rinsed in 1X PBS for 5 min twice, then coverslipped using Prolong Gold antifade with DAPI. All slides were stored protected from light until analysis using a Nikon Eclipse E800 epifluorescence microscope equipped with a Nikon Digital Camera DXM 1200 or an Olympus Fluoview FV 1000 confocal.

### TUNEL labeling

Frozen tissues samples were allowed to warm to room temperature for 10–15 min and subjected to TUNEL labeling using an *In situ* cell death detection kit (Roche, Indianapolis, IN) as per manufacturer's instructions. Briefly, tissue sections were post-fixed with 4% paraformaldehyde, washed with 1X PBS for 30 min at room temperature and permeabilized with 0.1% Triton X-100, 0.1% sodium citrate in 1X PBS for 2 min on ice. Sections were then rinsed with 1X PBS, followed by incubation with TUNEL reaction mixture for 60 min at 37°C in a humidified atmosphere in the dark. Slides were then rinsed 3 times with 1X PBS and mounted using an antifade agent (Prolong Gold, Invitrogen, Carlsbad, CA). Samples were analyzed using an Olympus confocal laser scanning microscope using an excitation wavelength of 488 nM. A negative control without addition of label solution and a positive control incubated with 3000 U/ml of DNAase I recombinant were also subjected to similar conditions and analyzed.

### Tissue Analysis

Digital images for cell death (TUNEL and caspase 3) and retinal cell layer thickness measurements (H+E stain) were taken by an Olympus Fluoview FV1000 confocal microscope. For cell death, positively labeled cells were counted over a 100 µm area that was 200 µm dorsal and ventral from the optic nerve. Similar to cell death assays, we measured retinal cell layer thicknesses 200 µm dorsal and ventral of the optic nerve using the FV10-ASW 2.1 Viewer software of the Olympus Fluoview FV1000 confocal microscope. To determine the significance of our data, we used an unpaired t-test that compared age-matched littermates of wild type (WT) and mutant rats (Graphpad Software, http://www.graphpad.com/quickcalcs/ttest1.cfm).

### Transmission Electron Microscopy

Wpk rats were perfused through the left ventricle with 4% paraformaldehyde in 0.1 M phosphate buffer. Tissue sections used for electron microscopy were placed in 2% paraformaldehyde, 2% glutaraldehyde in phosphate buffer. Tissue was processed for TEM by the Electron microscopy Center at the Indiana School of Medicine, Indianapolis using standard methods (http://anatomy.iupui.edu/core-facilities/electron-microscopy-center/). Briefly, tissue was cut into 1×2 mm segments, post-fixed in osmium tetroxide, dehydrated in a graded series of ethanol, infiltrated and embedded in Embed 812 (Electron Microscopy Sciences). Sections were cut with a diamond knife on a Leica UCT Ultramicrotome (Leica), stained with uranyl acetate and examined using a Tecnai G2 12 Bio Twin (FEI) [22].

## Results

### Meckelin 3 is found in developing and mature rat retina

**Figure 1:**
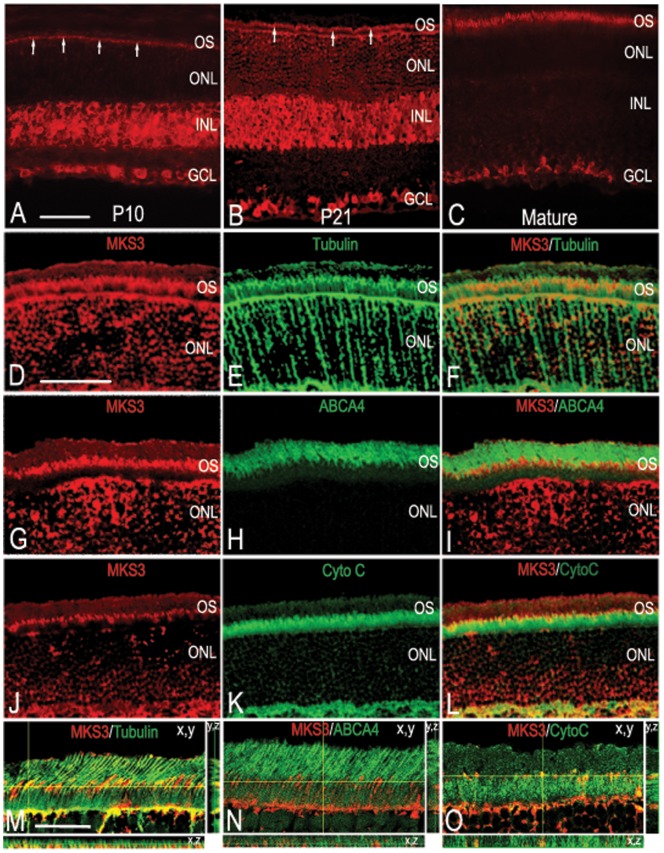
Meckelin 3 in the developing rat retina. Confocal imaging of wild-type P10 (A), P21 (B), and mature (C) retinal sections immunolabeled with antibody specific for MKS3 showed that MKS3 expression was widespread in the early postnatal retina that becomes restricted in the mature retina. (A) At P10 MKS3 could be detected in the region of the ONL consistent with developing inner and outer segments (arrows), as well as the inner and ganglion cell layers. (B) At P21, MKS3 showed similar localization in comparison to P10, but developing outer segments were much more visible at this stage (arrows). (C) MKS3 label was detected in photoreceptor outer segments and ganglion cells; no label in inner nuclear was detected at this stage. Retinal sections were also double-labeled with MKS3 (D,G, J) and tubulin (E), ABCA4 (H), or cytochrome C (K) to better localize MKS3 protein within the photoreceptor inner and outer segments. An overlay of MKS3 and tubulin (F) highlighted the localization of MKS3 in the photoreceptor axoneme, which spans the inner segment, connective cilium, and the lower portion of the outer segment. Consistent with the localization of MKS3 within the axoneme, there was a partial overlap with cytochrome C (L) within the upper portion of the inner segment. There appears to be little to no overlap with the outer segment marker, ABCA4 (I), indicative of MKS3 localization primarily to the connective cilium and lower portion of the outer segment. Thin plane confocal microscopy on sections labeled for MKS3 and tubulin (M), ABCA4 (N), or Cytochrome C (O) in order to verify patterns of expression. For M, N, and O, the xy, planes are labeled and the yellow lines indicate the x,z and y,z planes depicted in the strips to the right and the bottom. GCL, ganglion cell layer; INL, inner nuclear layer; ONL, outer nuclear layer; OS, outer segment; CytoC, cytochrome C; ABCA4, ATP-binding cassette sub-family A – member 4. Scale bar: (A) Bar  = 50 µm for panels A–C, (D) Bar  = 50 µm for panels D–L, (M) 100 µm for M–O.

To determine the localization of MKS3 in the developing rat retina, immunohistochemistry was performed at three stages critical for photoreceptor outer segment development; postnatal day 10 (P10) when many photoreceptors have begun to make rudimentary outer segment processes, P21 when OS are mature and 6 weeks when the retina is fully mature. At earlier stages, expression of MKS3 appeared to be fairly ubiquitous (Fig 1). At P10 MKS3 was found in the region where inner and OS are being formed in the outer nuclear layer (arrows; Fig 1A), as well as the inner and ganglion cell layers. At P21, both the inner and ganglion cell layers were still uniformly labeled, and MKS3 could clearly be detected in the presumptive mature photoreceptor outer segment (Fig 1B). In contrast, MKS3 was detected only in the photoreceptor inner/outer segments and ganglion cells of the mature retina (Fig 1C). Staining was no longer detectable in the INL. No staining was detected in sections of retina from the bilateral polycystic kidney mouse (BPCK), in which a large region of chromosome 4 that includes the MKS3 gene has been deleted (not shown). Negative controls in which sections of retina were treated with IgG in place of primary antibody also showed no label (not shown).

To examine the subcellular distribution of meckelin within the photoreceptor inner and outer segment, we double-labeled cryosections through P21 retina for MKS3 and antibodies against proteins known to be present primarily in the axoneme (tubulin), the inner segment (Na^+^/K^+^ ATPase α3), or the outer segment (ATP binding cassette subfamily A4; ABCA4) [23], [24]. MKS3 co-localized to a great extent with tubulin (Fig 1D–F) and partially with cytochrome C (Fig 1J–L), but appeared not to co-localize with ABCA4 (Fig 1G–I). Co-localization was verified by double-labeling sections and using thin-plane confocal microscopy in xy, xz, and yz planes (Fig M–O). The overlap in expression patterns with known markers indicated that MKS3 was localized to the inner segment and axoneme.

**Figure 2:**
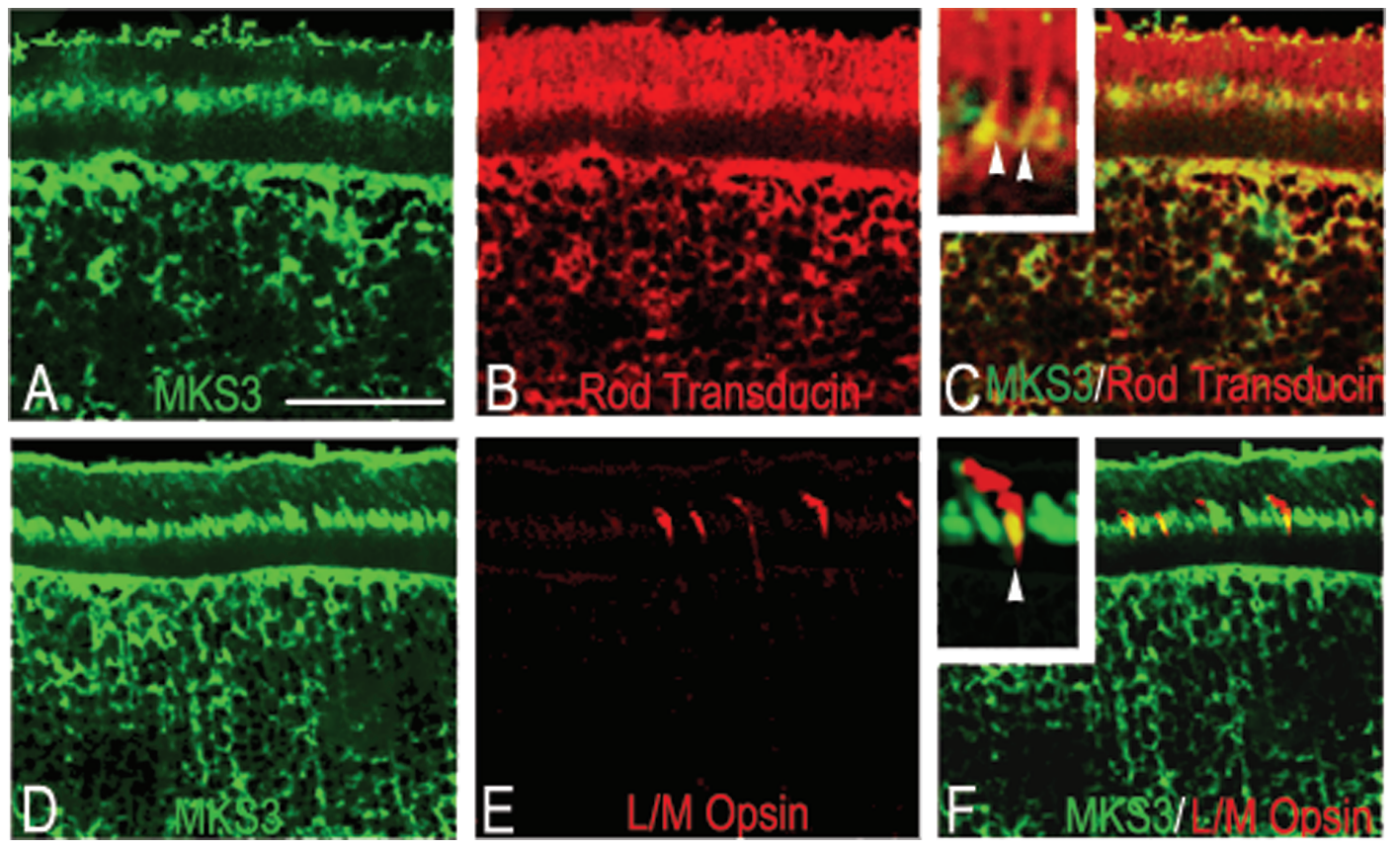
MKS3 was found in rods and cones. Double-label immunohistochemistry was performed on sections through P21 WT retina using an antibody specific for MKS3 (A, D) and rod transducin (B) or L/M opsin (E). Confocal images were then merged into a single image (C, F) to show the co-expression of MKS3 and photoreceptors in the outer segment. Increased magnification in insets (C, F) indicated there is only partial overlap in localization of both rod transducin and L/M opsin with MKS3. GCL, ganglion cell layer; INL, inner nuclear layer; ONL, outer nuclear layer; OS, outer segment. Scale bar: (A) 50 µm.

To confirm that MKS3 was expressed in all retinal cell types, we performed double-label immunohistochemistry using antibodies against specific retinal cell markers at P21. The expression of meckelin in rods and cones was first determined (Fig 2). MKS3 was clearly detected in both photoreceptor cell types labeled as seen in overlays of sections co-labeled for rod transducin and meckelin (Fig 2A–C) or long/medium opsin and meckelin (L/M opsin; Fig 2D–F). In both cases, there was only partial overlap in the localization of the MKS3 with both transduction molecules. MKS3 appeared to ubiquitously label cells of the inner nuclear and ganglion cell layers. Cells co-expressed MKS3 and calbindin (horizontal cells; Fig S1A–C), Chx10 (bipolar cells; Fig S1D–F), glutamine synthetase (Müller glia and retinal astrocytes; Fig S1G–I), parvalbumin (amacrine cells; Fig S1J–L), and Brn3a (ganglion cells; Fig S1M–O).

### MKS3 co-localizes with IFT20

**Figure 3:**
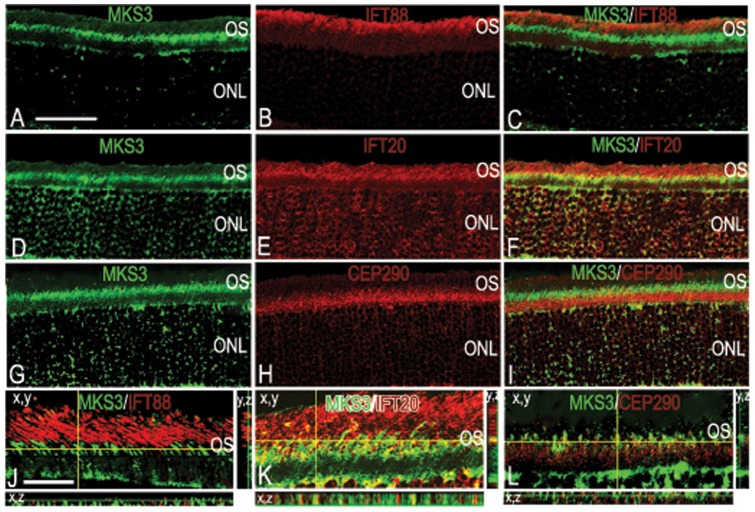
MKS3 showed partial co-localization with IFT20. To elucidate which IFT proteins might interact with MKS3, we performed double-label immunohistochemistry with MKS3 (A, D, G) and IFT88 (B), IFT20 (E), and Cep290 (H). Overlay of confocal images (C, F, I) showed that MKS3 was partially co-localized with IFT 20 (F). MKS3 was not co-localized with IFT88 (C), or Cep290 (I). Localization data was verified using thin-plane confocal microscopy (J–L). In J–L, the horizontal yellow line in the x,y image indicates the focal plane illustrated in the x,z strip at the bottom of the panel and the vertical yellow line in x,y image indicates the focal plane illustrated in the y,z panel. Scale Bar  = (A) 50 µm for panels A-I and (J) 100 µm for panels J–L.

In order to distinguish with which IFTs MKS3 might interact, we chose 3 proteins that are found in different parts of the rat intraflagellar transport process from through the Golgi complex, inner segment, basal connective cilium (IFT20), the basal body (Cep290), and in outer segment discs (IFT88) [4], [25], [26]. MKS3 appeared not to co-localize with either IFT88 (Fig 3A-C) or Cep290 (Fig 3G-I), but showed partial co-localization with IFT20 (Fig 3D-F). Co-localization or lack thereof wit IFT88, IFT20, and Cep290 were verified using thin-plane confocal microscopy in xy, xz, and yz planes (Fig 3J–L). MKS3 label is apparent in the inner segment and possibly the basal portion of the connecting cilium in agreement with the pattern of co-localization patterns with tubulin and cytochrome C (compare Fig 3 with Fig 1D–F and J–L).

### Cell loss in WPK mutant retinae

To further test the hypothesis that MKS3 is critical for the formation of photoreceptor OS, we utilized a rat with a spontaneous autosomal recessive mutation of the *Mks3* gene, referred to as the rat Wistar polycystic kidney disease model (WPK) [27]. We restricted our analysis to stages between P10 and P21 for two reasons. First, earlier analyses by Tammachote et al., [20] indicated that the photoreceptors were capable of forming an axonemal structure, hence we reasoned that the early stages of outer segment development were likely not to be affected (albeit they may not form the structures on the same time scale as their wild type counterpart). Since the outer segment axoneme begins extending in some photoreceptors at around P5 in the rat, we decided on a slightly later stage of P10 at which stage outer segment development beyond the axoneme is evident [28], [29]. Second, the progression of the polycystic kidney disease (PKD) has been well characterized in this strain of rats and culminates in a majority of the rats dying between 4 and 6 weeks of age [27], [30]. Therefore we restricted our analysis of the mature retinae to P21, prior to the period when rats were extremely ill and/or dying.

**Figure 4:**
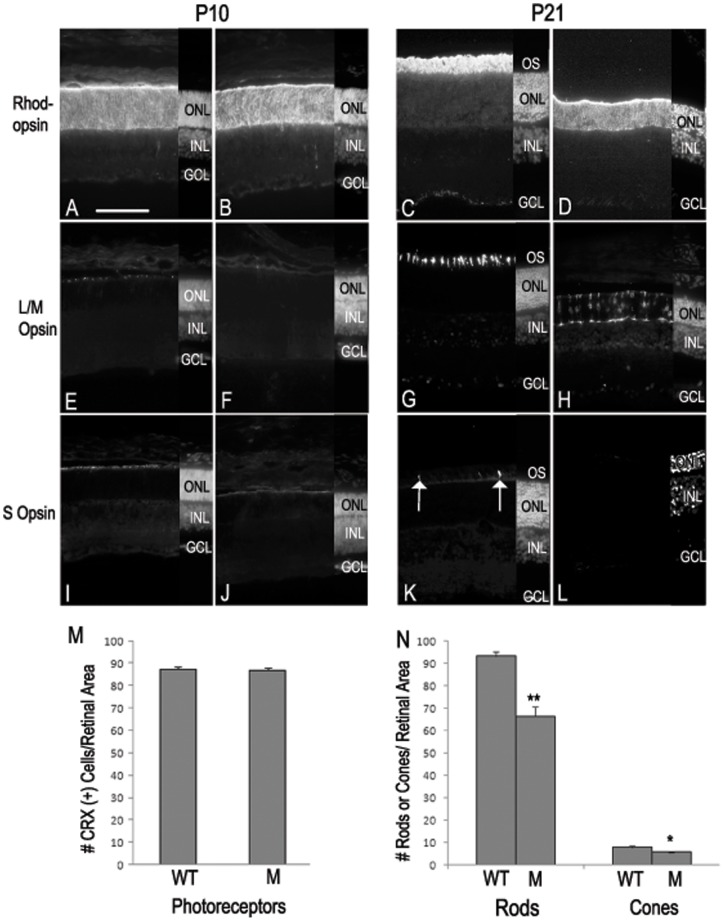
Loss of photoreceptors between P10 and P21 in MKS3 mutant. To determine the effect of *Mks3* mutation in photoreceptors at P10 and P21, sections were immunolabeled for rhodopsin (A–D), L/M opsin (E–H), or S opsin (I–L). At P10, rhodopsin-labeled cells were easily detected in seemingly similar numbers in the WT and mutant retinae (A, B). Both L/M opsin- and S opsin-labeled cells were not abundant at P10 in either the WT or mutant retinae (E, F and I, J). By P21, rhodopsin was highly abundant in the photoreceptor outer segments of WT retinae, but was present in the cell bodies of the photoreceptors of mutant retinae (C, D). Similarly, outer segments labeled for L/M opsin were easily detected in WT photoreceptors, but staining was localized to the cell body of mutant photoreceptors (G, H). S opsin-positive cells were detectable in WT photoreceptor outer segments, but very few positive cells were detected in mutant retinae (arrow; K, L). DAPI label of the section is shown in a small strip on the right-hand side of each picture to indicate placement of the retinal cell layers (A–L). Quantitatively, the number of CRX (+) cells was very similar in WT and mutant retinae at P10 (M). However, there was a large drop in the number of rods and a smaller, but statistically significant decrease in the number of L/M cones was found in the fundus of the mutant retinae in comparison to the WT (N). GCL, ganglion cell layer; INL, inner nuclear layer; ONL, outer nuclear layer; OS, outer segment; WT, wild type; M, mutant. ** unpaired t-test p>0.005. * unpaired t-test p>0.05. Scale bar: (A) 50 µm.

To analyze cell loss that appeared to occur in the mutant, we first immunolabeled cryosections from control WT and mutant P10 and P21 eyes with antibodies specific for rhodopsin (rods), L/M opsin (cones), and S opsin (cones) in the fundus of the retinae (Fig 4). While opsin labeling at P10 appeared similar in WT and mutant retinae (Fig 4A, B, E, F, I, J), it was apparent that at P21 both rods and cones were severely affected by the MKS3 mutation (Fig 4C, D, G, H, K, L). In comparison to age-matched WT littermates, there appeared to be a significant reduction in the number of labeled rods at P21, and labeling that was retained was localized to the rod cell bodies, rather than the label in the outer segment process of WT photoreceptors (Fig 4C, D). Further, immunolabeling for the L/M opsin in mutant retinae revealed mislocalization of the opsins to cell bodies in comparison to the WT control which showed labeling in the inner and outer segment (Fig 4G, H). Very few cells were S opsin-positive in the WT retina (Fig 4K) and none could be found in the WPK mutant (Fig 4L). A quantitation of the number of photoreceptors at P10 and P21 confirms the immunohistochemical observations. Since L/M and S opsin labels very few cells at P10, the number of cells expressing cone-rod homeobox (CRX) protein was quantitated. At P10, the number of CRX^+^ cells in WT and mutant retinae were nearly identical (Fig 4M). At P21 the number of rods was quantitated by counting the number of recoverin^+^ cells, while the number of L/M cones was quantitated by counting L/M^+^ cells. Counts at P21 revealed that there was an approximate 30% drop in the number of rods in the mutant retinae in comparison to WT and a small but statistically significant decrease in the number of L/M cones (Fig 4N). S cones were not counted.

To investigate whether there was a loss of any of cells in the inner and ganglion cell layers, we immunolabeled P10 (not shown) and P21 cryosections with calbindin (horizontal cells; Fig S2A, B), Chx10 (bipolar cells; Fig S2C, D), Sox2 (Müller glia and astrocytes; Fig S2E, F), parvalbumin (amacrine cells; Fig S2G, H), or Brn3a (ganglion cells; Fig S2I, J). At both P10 and P21 there did not appear to be any significant alterations in any of the markers examined. Counts of each cell type in the fundus of the retinae at P10 and P21 were nearly identical, indicating there was little or no cell loss in the inner and ganglion cell layers of the mutant retinae (Fig S2M, N). Since there was considerable photoreceptor cell loss at P21, we determined whether there might also be reactive gliosis as a result of the cell loss. To detect reactive gliosis, the WT and mutant sections were immunolabeled with an antibody-specific for glial fibrillary acidic protein (GFAP). A small amount of GFAP expression was localized to the Müller glial endfeet at the inner and outer limiting membranes and retinal astrocytes in WT retinae (Fig S2K); however, a significant increase of GFAP occurred throughout the cell body of Müller glia and retinal astrocytes of the mutant (Fig S2L).

**Figure 5:**
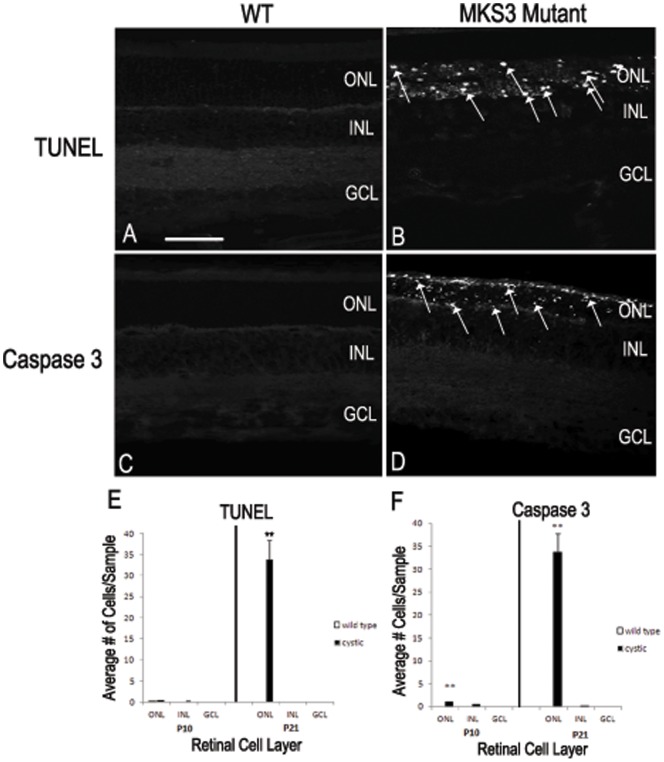
Cell death of MKS3 mutant photoreceptors. The amount of cell death was observed by TUNEL and caspase labeling of the P21 WT (A, C) and P21 mutant (B, D) retinae. Both TUNEL and caspase 3 labels showed an abundance of cell death expression in the outer nuclear layer of the P21 mutant rat retinae in comparison to the WT (arrows; B, D). Graphs depict counts of cells positive for TUNEL (E) and caspase 3 (F) label in each of the retinal cell layers (n = 3 retinae for each sample). There were no significant differences in the number of TUNEL (+) cells at P10; however, there was a large statistically significant increase in the number of TUNEL (+) cells at P21 (p<0.005). No significant differences were noted in the WT and mutant INL and GCL at P10 and P21 using caspase 3. However, statistically significant increases in caspase 3-labeled cells were found in the ONL of the *Mks3* mutant in comparison to WT at both P10 and P21. (*p<0.01 and **p<0.001 respectively). GCL, ganglion cell layer; INL, inner nuclear layer; ONL, outer nuclear layer; OS, outer segment. Scale bar: (A) 50 µm.

To analyze the apparent decrease in photoreceptors in the WPK mutants, we labeled sections through central retina with the terminal deoxynucleotidyl transferase mediated dUTP nick-end-labeling (TUNEL) assay or with an antibody against caspase 3 to detect cells undergoing apoptosis. At P10, there were very few cells labeled with TUNEL or caspase 3 in the INL and GCL of the WT or MKS3 mutant retinae (not shown). Similarly, at P21 there were also very few TUNEL and caspase 3 positive cells in the retinae of control WT animals (Fig 5A, C). However, an analysis of P21 mutant retinae indicated that there were a substantial number of both TUNEL- and caspase 3-labeled cell in the ONL in comparison to WT (Fig 5B, D). The INL and GCL of P21 mutant retinae appeared to be similar to the WT in that very few cells were labeled with TUNEL or caspase 3. Quantitation of WT and mutant retinae in the fundus at both P10 and P21 showed similar results to those of the qualitative results above; TUNEL-labeled cells were sparse in both mutant and WT P10 sections, while there were approximately 33 cells per sample labeled in the ONL of the mutant retina (Fig 5E). However in caspase 3-labeled sections, a small but statistically significant increase in the number of cells were found in the mutant retinae at P10 in comparison to WT (Fig 5F). At P21, there were a similar number of caspase 3-labeled cells in the ONL of the mutant retinae as were found in the TUNEL-labeled sections. The increase in the number of both TUNEL and caspase 3-labeled cells were very similar to the decrease in the number of cells in the ONL found when counting opsin positive cells (compare Fig 5E, F with Fig 4N).

### IFT88, IFT20, and Cep290 localize to the inner and axonemal region in the MKS mutant

**Figure 6:**
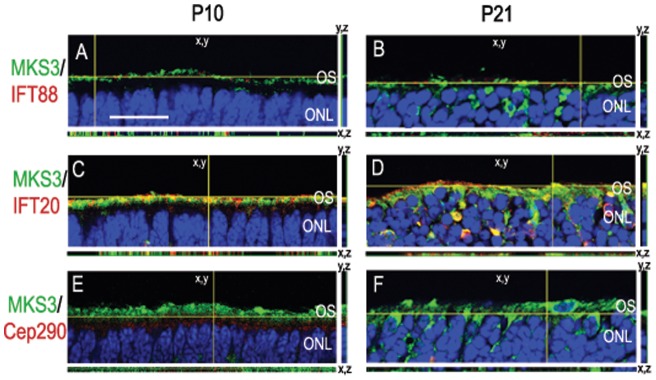
Localization of MKS3, IFT88, IFT20, and Cep290 in MKS3 mutant. Sections of P10 (A, C, E) and P21 (B, D, F) MKS3 mutant retina were double-labeled for MKS3 and IFT88 (A, B), IFT20 (C, D), or Cep290 (E, F) and analyzed with thin-plane confocal microscopy. The horizontal yellow line in the x,y image of each panel indicates the focal plane illustrated in the x,z strip at the bottom of the panel and the vertical yellow line in x,y image indicates the focal plane illustrated in the y,z panel. Blue label in all panels is DAPI to highlight the localization of the nuclei. (A) Scale Bar  = 100 µm.

Since L/M opsin and rhodopsin were mislocalized to the photoreceptor cell body in mutant retinas, it was of interest to determine if the MKS3 or other proteins involved in outer segment genesis were also aberrantly localized. Sections through P10 and P21 MKS mutants were double-labeled for MKS3 and IFT88 (Fig 6A, B), IFT20 (Fig 6C, D) or Cep290 (Fig 6E, F) and analyzed using thin-plane confocal microscopy. Unlike the opsins, MKS3, IFT20, IFT88, and Cep290 appeared to be localized to the inner segment/axonemal region of the photoreceptors at P10 (Fig 6A, C) and P21 (Fig 6B, D). Cep290 was localized to the inner segment below the regions where MKS3 was found at P10 (Fig 6E). By contrast, at P21 very little Cep290 was detected in the inner segment or the cell body (Fig 6F). As was seen in wild type retina (Fig 3), there was considerable co-localization of MKS3 and IFT20 was detected in the P10 mutant retina (Fig 6C), but less expression of IFT20 was evident at P21 (Fig 6D). No co-localization was evident with MKS3 and IFT88 or Cep290 at either P10 or P21 (Fig 6A, B, E, F).

### Rudimentary outer segments were initiated prior to photoreceptor degeneration in the WPK mutant

**Figure 7:**
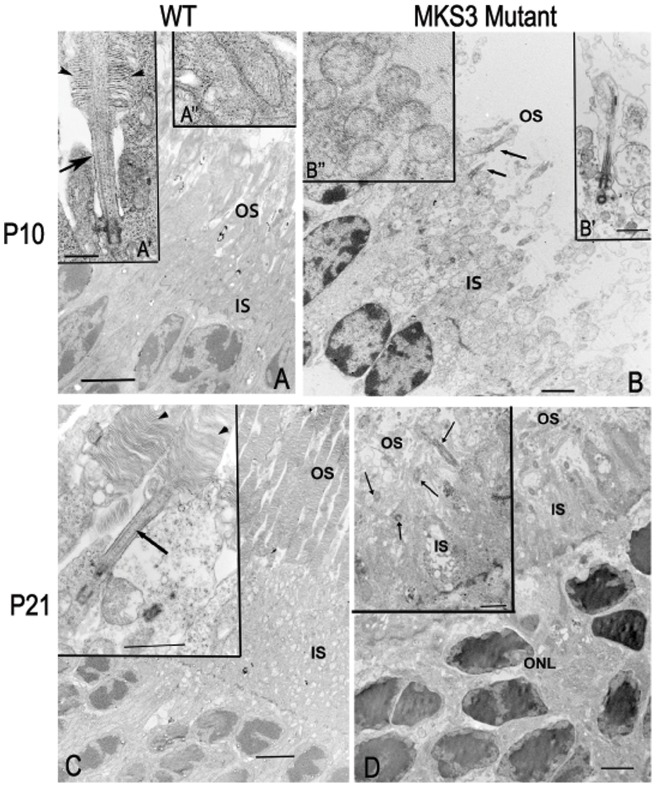
Electron microscopy of photoreceptor outer segments. Electron microscopic images were compared at P10 and P21 in WT (A, C) and mutant (B, D) retinae. At P10, the nascent outer segment discs can be found in WT (A', arrowhead) while in the mutant (B, arrows) the outer segment was not apparent, but a membranous loop without discs was associated with the cilium. Mitochondria in WT P10 appeared as elongated structures with visible cristae (A’’), while in the mutant retina the mitochondria appeared swollen with no evidence of cristae (B’’). At P21, the WT retina has a well-developed OS with discs (C), while the mutant (D) demonstrates cilia extending into the OS space with some loosely associated membranous material, but no discs were observed (inset, D). (A, C) IS; inner segments, OS; outer segments, WT; wild type. Scale Bar  = 4 µm, (B, D) 2 µm; Insets for A  = 500 nm, B- D  = 1 µm.

In the WT rat model, photoreceptor OS begin to develop at around P5 and fully mature by 3 weeks [28]. To determine if photoreceptor OS initially form in the WPK rat, transmission electron microscopy (TEM) images were compared in the control WT retina and the WPK rat retina. High resolution TEM images show inner and outer segment formation at P10 and P21 in WT and WPK rats (Fig 7). At P10, rods in the WT retinae were just beginning to form OS accompanied by laminated discs (Fig 7A'). However, the WPK mutant photoreceptor cilia form bulbous terminae without the formation of discs (Fig 7B'). In addition, mitochondria show swelling and breakdown of cristae (Figure 7B’’) in comparison to wild type (Fig 7A’’). By P21, the WT retinae have completed outer segment formation with numerous laminated discs (Fig 7C with inset). However, in the retinae of mutant rats, photoreceptors lack OS with the cilia projecting into a loosely organized area without evidence of laminated discs or intact mitochondria (Fig 7D inset). In the mutant areas of vacuolated membrane are seen in the inner segments at both P10 and P21. At P21 the photoreceptor nuclei have started apoptotic chromatin condensation (Figure 7D).

## Discussion

In this study, we have investigated the normal expression patterns of the MKS3 protein in the developing rat retina and tested its role in outer segment formation. The following summarizes our findings: 1) MKS3 was ubiquitously expressed in the P10 and P21 rat retina, 2) MKS3 was restricted to the ONL and GCL of the mature rat retina, 3) within photoreceptors, MKS3 was localized primarily to the distal inner segment, axoneme, and cell body, 4) rats with a naturally occurring point mutation in the *Mks3* gene showed a loss in the thickness of the ONL as well as an apparent loss in the number of cells labeled with rod and cone-specific markers in comparison to the WT, 5) the loss of OS in photoreceptors was linked to an increase in apoptosis, and 6) EM data showed that WT and WPK photoreceptors developed an axoneme on the same time scale; however MKS3 mutant rats were only able to produce rudimentary outer segment. We conclude from these studies that the MKS3 protein product is essential for the development of the outer segment in both rods and cones and the absence of the outer segment process leads to apoptosis of the developing rods and cones.

### Photoreceptor outer segment development and meckelin 3

The OS of the photoreceptor is initially developed as an extension of the connecting cilium. The basal side of the connecting cilium is anchored in the inner segment by the basal body and its apical side elongates to form the start of the outer segment. The stacked disks of the outer segment that house the visual pigments necessary for phototransduction are formed through a process that is still somewhat controversial. The disks arise from either the evagination of the plasma membrane from the base of the connecting cilia or the fusion and compression of pinocytic vesicles that arise from the plasma membrane [31]–[35]. In vertebrate rod cells, these disk membranes pinch off and separate, while in cone cells they are continuous with the plasma membrane. This outer segment disk formation is continued throughout adulthood due to the daily shedding of the disks from the most apical end of the OS [36]. In the studies presented here, rudimentary rod photoreceptor OS consisting of the connecting cilium and plasma membrane were present. However, no further outer segment development was noted. It is possible that outer segment discs may have developed between P10 and P21, and then subsequently degenerated. While we cannot rule this out completely, hematoxylin and eosin stained sections from P14 retinae indicate that no OS had been generated by that point and appeared essentially identical to the P10 retina (unpublished data). In addition, EM examination shows mitochondrial degeneration at P10, inconsistent with any further photoreceptor development.

### MKS3 localization in the retina

Our immunolabeling data indicated that within the photoreceptor, MKS3 is localized to the cell body, distal inner segment, and axoneme. The lack of staining of the BPCK mouse retina that lacks the *mks3* gene increases the reliability that the staining we describe is meckelin 3. Two pieces of data described herein lead us to believe that MKS3 is localized to the juncture of the inner and outer segments. First, double-label experiments showed that MKS3 overlapped with tubulin (connective cilium and inner segment) at the basal edge and cytochrome C (inner segment) at the apical edge (Fig 1). This places MKS3 above most of the mitochondria in the inner segment and below the majority of the connective cilium. Experiments in which we double-labeled with MKS3 and Cep290 (basal body), IFT20 or IFT88 were in agreement with MKS3 localization to the base of the connective cilium. Overlap in label was detected with IFT20, a transport protein located at the basal portion of the connective cilium [4]. Overlap of MKS3 with IFT20 was found at the basal-most region of the IFT20 label (Figure 3). This data also places the MKS3 at the basal-most portion of the connective cilium. Others have shown that MKS3 interacts with family members MKS1 and 2, both of which have also been localized to the transition zone non-photoreceptor cilia [18], [37]–[39]. We propose that MKS3 is localized to the periciliary region of the photoreceptor, which is the equivalent to the transition zone in non-photoreceptor cilia [40].

Other studies have localized MKS3 at or near the basal body which we did not confirm in our studies [19]. There are a few possible explanations for this discrepancy: 1) Adams et al., [19] used an MKS3 antibody that recognizes an epitope on the N-terminus of the protein, whereas the antibody used in this study recognizes a C-terminus epitope. It is possible that the C-terminus epitope is not accessible when MKS3 was localized to the basal body. 2) Perhaps the Cep290 and MKS3 are localized to separate and distinct regions of the basal body. 3) There may be subtle and undescribed differences in the MKS3 localization in photoreceptors and kidney cells.

### Cell death in MKS3 mutants

There was substantial cell death associated with photoreceptors in the MKS3 mutant rat by postnatal day 21. Photoreceptor death appears to be a consistent theme in animal studies as well as in human diseases involving retinal disease [41]–[44]. Photoreceptors appear to preferentially use the caspase-dependent apoptotic pathway during degeneration [45]. We initially tested for cell death using the TUNEL assay, which is a non-specific marker of cell death occurring by apoptotic as well as necrotic and autolytic pathways [46], [47]. We subsequently showed using the localization of a member of the apoptotic pathway, caspase 3, that the photoreceptors in the mutant rat were using the caspase-dependent pathway [48], [49]. The mitochondrial deterioration shown by EM examination is consistent with activation of the caspase-dependent apoptotic pathways [50].

Just as interesting as the presence of photoreceptor degeneration is the apparent absence of degeneration in other cell types in the mutant retina. In addition to the inner and outer segment of WT retinae, MKS3 was also localized to Müller, bipolar, horizontal, amacrine, and ganglion cells of the developing retina. While these cells express the MKS3, they appear not to undergo degeneration, at least during the period examined in the studies presented here. Barring the possibility that the cells degenerate later in development, perhaps the most logical reason these cells do not degenerate is that the cilia may be a vestigial apparatus in many cells [51]. Primary cilia are present in almost all cells, but they appear to have a function only in a subset of those cells [52]. Alternatively, cilia in these other cells may have an as yet undescribed function, but the cells are not as reliant upon this particular function for their survival [53]. Further study is needed to clarify the potential contribution(s) of MKS3 in other retinal cell types.

### Comparison of MKS3 mutants with other models of photoreceptor degeneration

An analysis of the literature indicates photoreceptor degeneration in mammalian retina upon loss of IFT–related genes; 1) early defects in the outer segment elongation or organization that leads to early and progressive photoreceptor degeneration [25], [54]–[65], and 2) fairly normal development of outer segments, followed by slow, progressive photoreceptor degeneration [66], [67]. Further, within the early progressive group, there are animal models that developed abnormal outer segments that then degenerated, such as RP1 mutants, and other models in which only a nascent connective cilium developed [55], [57]. The mutation described in this study resembled models in which there was fast progressive degeneration with no development of an outer segment beyond the nascent connective cilium. Clearly, IFT or IFT-related proteins are necessary for the development and/or maintenance of outer segments; however, it is unclear why a lack of outer segment would lead to photoreceptor degeneration. A number of hypotheses have been investigated in regards to potential causes of photoreceptor apoptosis that occurs in with outer segment dysmorphogenesis, including 1) endoplasmic reticulum stress brought about by excessive proteins localized to the cell body, 2) constitutive activation of mislocalized opsin proteins, 3) stimulation of somal signaling pathways that normally are inaccessible to opsin, and 4) an increase in reactive oxygen species resulting from a lack of oxygen usage by dysfunctional outer segments [45], [68]–[70]. In the developing rat retina, opsin protein was detectable by P2 in the retinal fundus and increased thereafter to reach adult levels [71]. We detected opsin expression at P10 in the MKS3 mutant retina, the earliest stage examined; hence the mislocalization of opsin protein may play a role in photoreceptor death in the MKS3 mutant. In regards to the timeline of apoptosis in the MKS3 retina, TUNEL labeled cells were not detectable at P10, but a few caspase-labeled cells were observed, indicating that the degenerative process was already underway in the cells at that point. By P21, the latest timepoint we examined in this study, over one third of the photoreceptors had degenerated.

The potential role of opsin mislocalization in photoreceptor degeneration suggests that light may modify the degenerative process. The potential effect of reducing photoreceptor degeneration by reducing light exposure has been tested on various animal models and in human disease states with mixed results. Photoreceptor degeneration can be ameliorated in a number of animal models by rearing animals in the dark [72]–[83]. However, there are also multiple photoreceptor degeneration-causing mutations that are not affected by dark-rearing [74], [84]–[91]. As discussed by Valter and colleagues [80], there are a number of variables that can have a significant impact on whether dark-rearing ameliorates photoreceptor degeneration. For instance, genetic background, time of day that the light exposure is given, prior history of light exposure, duration and strength of exposure, and age of animal can all alter responsiveness of photoreceptors to changes in lighting. The typical paradigm used to reduce photoreceptor degeneration in animal models is to rear animals in the dark from an early age Evidence from the Stone lab suggests an alternate paradigm: treatment of developing photoreceptors with a priming light stimulus may in fact lead to increased photoreceptor survival in the adult, whereas early light restriction actually makes adult photoreceptors more susceptible to degeneration [80]. The possibility that photoreceptor degeneration might be decreased by altering light exposure has not been tested with the MKS3 mutation, but is an attractive possibility for non-invasive treatment.

## Supporting Information

Figure S1 **MKS3 is widely expressed in the P21 WT rat retina.** Double-label immunofluorescence was performed in P21 retina with MKS3 (A, D, G, J, M) and calbindin (horizontal cells; B), Chx10 (bipolar cells; E), glutamine synthetase (Müller glia and retinal astrocytes; H), parvalbumin (amacrine cells; K) or Brn3a (ganglion cells; N). Co-expression of meckelin with cell type-specific markers can be seen in C, F, I, L, and O. MKS3 appeared to be co-localized with cell type-specific markers found in the ONL, INL, and GCL. GCL, ganglion cell layer; INL, inner nuclear layer; ONL, outer nuclear layer; Calb, calbindin; GS, glutamine synthetase; PV, parvalbumin. Scale bar: (A) 50 µm.
(TIF)

Figure S2 **No apparent loss of INL or GCL cells in MKS3 mutant.** Cell type-specific immunolabeling was performed using sections from P21 WT (A, C, E, G, I, K) and *Mks3* mutant (B, D, F, H, J, L): horizontal cells were labeled with calbindin (A, B), bipolar cells with Chx10 (C, D), Müller glia cells with Sox2 (E, F), amacrine cells with parvalbumin (G, H), and ganglion cells with Brn3a (I, J). Calbindin, Chx10, Sox2 and Brn3a positive cells were fairly similar in number in WT and mutant retinae. A similar number of parvalbumin (+) cells were found in both the WT and mutant retinae, but with a greater amount of those in the mutant were found in the GCL rather than the INL. Sections through WT (K) and mutant (L) retinae were labeled with glial fibrillary acidic protein to detect reactive glia present in degenerating retinae. There was little expression in the WT (K) at P21; however, there was a significant increase in the mutant (L). DAPI-label of the section is shown in a small strip on the right-hand side of each picture to indicate placement of the retinal cell layers (A–L). Graphs depict the average number of cells in inner and ganglion cell layers at P 10 (M) and P21 (N). GCL, ganglion cell layer; INL, inner nuclear layer; ONL, outer nuclear layer; Cal, calbindin; PV, parvalbumin; GFAP, glial fibrillary acidic protein. Scale bar: (A) 50 µm.
(TIF)
