# Supplementary figures and images for: Meckelin 3 Is Necessary for Photoreceptor Outer Segment Development in Rat Meckel Syndrome
Source: PLoS One. 2013 Mar 13;8(3):e59306. doi: 10.1371/journal.pone.0059306 (PMC3596335; doi:10.1371/journal.pone.0059306)

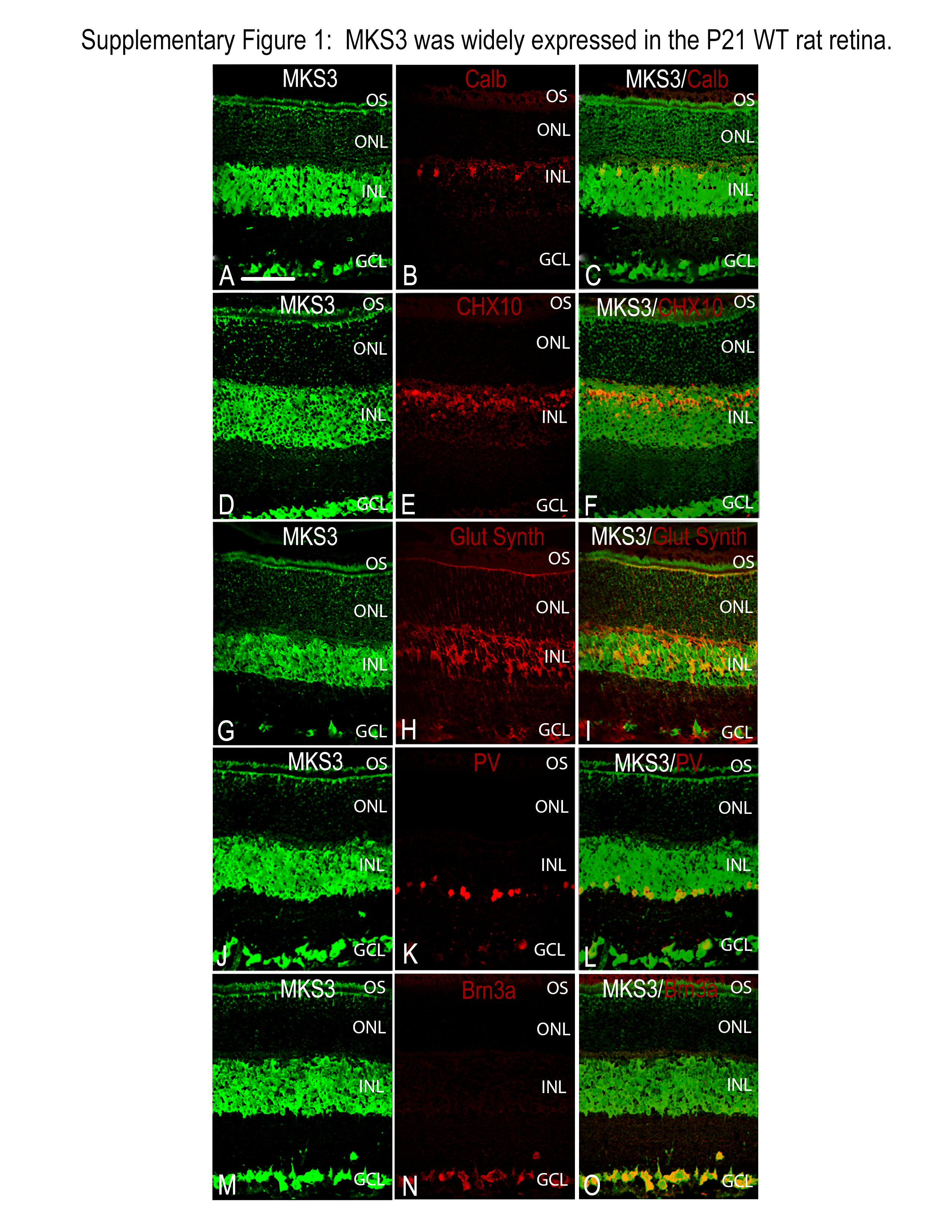

Supplement: Figure S1 — MKS3 is widely expressed in the P21 WT rat retina. Double-label immunofluorescence was performed in P21 retina with MKS3 (A, D, G, J, M) and calbindin (horizontal cells; B), Chx10 (bipolar cells; E), glutamine synthetase (Müller glia and retinal astrocytes; H), parvalbumin (amacrine cells; K) or Brn3a (ganglion cells; N). Co-expression of meckelin with cell type-specific markers can be seen in C, F, I, L, and O. MKS3 appeared to be co-localized with cell type-specific markers found in the ONL, INL, and GCL. GCL, ganglion cell layer; INL, inner nuclear layer; ONL, outer nuclear layer; Calb, calbindin; GS, glutamine synthetase; PV, parvalbumin. Scale bar: (A) 50 µm. (TIF) [file pone.0059306.s001.tif]

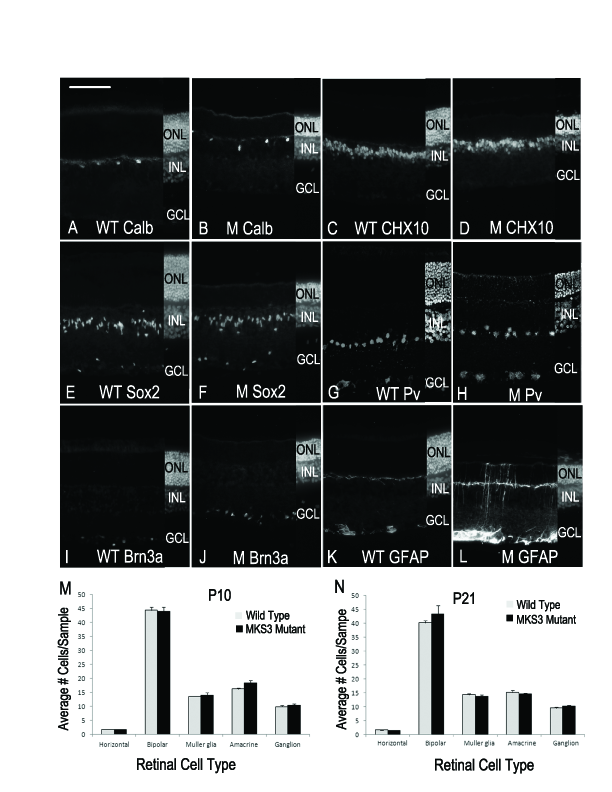

Supplement: Figure S2 — No apparent loss of INL or GCL cells in MKS3 mutant. Cell type-specific immunolabeling was performed using sections from P21 WT (A, C, E, G, I, K) and Mks3 mutant (B, D, F, H, J, L): horizontal cells were labeled with calbindin (A, B), bipolar cells with Chx10 (C, D), Müller glia cells with Sox2 (E, F), amacrine cells with parvalbumin (G, H), and ganglion cells with Brn3a (I, J). Calbindin, Chx10, Sox2 and Brn3a positive cells were fairly similar in number in WT and mutant retinae. A similar number of parvalbumin (+) cells were found in both the WT and mutant retinae, but with a greater amount of those in the mutant were found in the GCL rather than the INL. Sections through WT (K) and mutant (L) retinae were labeled with glial fibrillary acidic protein to detect reactive glia present in degenerating retinae. There was little expression in the WT (K) at P21; however, there was a significant increase in the mutant (L). DAPI-label of the section is shown in a small strip on the right-hand side of each picture to indicate placement of the retinal cell layers (A–L). Graphs depict the average number of cells in inner and ganglion cell layers at P 10 (M) and P21 (N). GCL, ganglion cell layer; INL, inner nuclear layer; ONL, outer nuclear layer; Cal, calbindin; PV, parvalbumin; GFAP, glial fibrillary acidic protein. Scale bar: (A) 50 µm. (TIF) [file pone.0059306.s002.tif]
